# Prognostic impact of obesity in newly-diagnosed glioblastoma: a secondary analysis of CeTeG/NOA-09 and GLARIUS

**DOI:** 10.1007/s11060-022-04046-z

**Published:** 2022-06-15

**Authors:** Johannes Weller, Niklas Schäfer, Christina Schaub, Anna-Laura Potthoff, Joachim P. Steinbach, Uwe Schlegel, Michael Sabel, Peter Hau, Clemens Seidel, Dietmar Krex, Roland Goldbrunner, Torsten Pietsch, Theophilos Tzaridis, Thomas Zeyen, Valeri Borger, Erdem Güresir, Hartmut Vatter, Ulrich Herrlinger, Matthias Schneider

**Affiliations:** 1 Division of Clinical Neurooncology, Department of Neurology, University Hospital Bonn, Venusberg-Campus 1, 53127 Bonn, Germany; 2 Department of Neurosurgery, University Hospital Bonn, Bonn, Germany; 3 Dr. Senckenberg Institute of Neurooncology, University of Frankfurt, Frankfurt, Germany; 4 Department of Neurology, University Hospital Knappschaftskrankenhaus, Ruhr–Universität Bochum, Bochum, Germany; 5 Department of Neurosurgery, University of Düsseldorf, Düsseldorf, Germany; 6 Department of Neurology and Wilhelm Sander NeuroOncology Unit, University Hospital Regensburg, Regensburg, Germany; 7 Department of Radiation Oncology, University of Leipzig, Leipzig, Germany; 8 Department of Neurosurgery, University of Dresden, Dresden, Germany; 9 Department of Neurosurgery, University of Cologne, Cologne, Germany; 10 Institute of Neuropathology, University Hospital Bonn, Bonn, Germany

**Keywords:** Glioblastoma, Temozolomide, Obesity, MGMT

## Abstract

**Purpose:**

The role of obesity in glioblastoma remains unclear, as previous analyses have reported contradicting results. Here, we evaluate the prognostic impact of obesity in two trial populations; CeTeG/NOA-09 (n = 129) for MGMT methylated glioblastoma patients comparing temozolomide (TMZ) to lomustine/TMZ, and GLARIUS (n = 170) for MGMT unmethylated glioblastoma patients comparing TMZ to bevacizumab/irinotecan, both in addition to surgery and radiotherapy.

**Methods:**

The impact of obesity (BMI ≥ 30 kg/m^2^) on overall survival (OS) and progression-free survival (PFS) was investigated with Kaplan–Meier analysis and log-rank tests. A multivariable Cox regression analysis was performed including known prognostic factors as covariables.

**Results:**

Overall, 22.6% of patients (67 of 297) were obese. Obesity was associated with shorter survival in patients with MGMT methylated glioblastoma (median OS 22.9 (95% CI 17.7–30.8) vs. 43.2 (32.5–54.4) months for obese and non-obese patients respectively, p = 0.001), but not in MGMT unmethylated glioblastoma (median OS 17.1 (15.8–18.9) vs 17.6 (14.7–20.8) months, p = 0.26). The prognostic impact of obesity in MGMT methylated glioblastoma was confirmed in a multivariable Cox regression (adjusted odds ratio: 2.57 (95% CI 1.53–4.31), p < 0.001) adjusted for age, sex, extent of resection, baseline steroids, Karnofsky performance score, and treatment arm.

**Conclusion:**

Obesity was associated with shorter survival in MGMT methylated, but not in MGMT unmethylated glioblastoma patients.

## Introduction

Despite recent therapeutic progress, glioblastoma remains a devastating disease with short survival [1]. Prognostic factors including age, Karnofsky performance scale (KPS), extent of resection, and MGMT promoter methylation status, aid to estimate the course of disease and enable shared decision-making regarding therapeutic options [2]. The impact of obesity on survival in high grade glioma has been retrospectively analyzed with contradicting results, as it was associated with better [3–6], indifferent [7], or worse survival [8, 9]. Notably, these studies exhibit limitations, including recruitment before current standard therapies [7], inclusion of different tumor grades [8, 9], the single- or bicentric retrospective nature of analyses [3–8], and not accounting for MGMT promoter methylation status.

Here, we aim to analyze the prognostic impact of obesity in glioblastoma with or without MGMT methylation using two well-characterized study cohorts.

## Methods

This study is a retrospective analysis of the prognostic impact of obesity in two prospective clinical trials of glioblastoma, which recruited at overlapping time periods at largely the same German university medical centers.

### CeTeG/NOA-09

This randomized phase III trial (ClinicalTrials.gov NCT01149109, [10]) included 129 patients aged 18–70 years with newly diagnosed glioblastoma, harboring a methylated MGMT promoter as determined by real-time methylation-specific PCR (msPCR [11]) and with a KPS of 70% or higher. Patients were recruited between June 2011 and April 2014 and randomized to standard temozolomide (TMZ) concomitant to radiotherapy followed by six courses of temozolomide or six courses of lomustine (CCNU) and TMZ starting during standard radiotherapy. As the study recruited before the 2016 WHO classification of tumours of the central nervous system, 23 patients with unknown IDH mutation status or confirmed IDH mutation were included [1].

### GLARIUS

This randomized phase II trial (ClinicalTrials.gov NCT00967330 [12]) included 170 patients aged 18 or older with newly diagnosed glioblastoma harboring an unmethylated MGMT promoter (same msPCR test as in CeTeG) and with a KPS of 70% or higher. Patients were recruited between June 2010 and August 2012 and randomized to standard TMZ concomitant to radiotherapy followed by six courses of TMZ, or standard radiotherapy with concomitant bevacizumab every 2 weeks followed by bevacizumab and irinotecan every 2 weeks.

### Statistical analysis

Descriptive statistics are provided as mean and standard deviation or median and interquartile range (IQR) where appropriate. Obesity was defined as a BMI of 30 kg/m^2^ or higher according to the WHO definition. Groupwise comparisons were performed using unpaired Student’s *t*-test, Wilcoxon rank-sum test or Fisher’s exact test, depending on scale and distribution. OS and PFS were analyzed with Kaplan–Meier analysis and log-rank test. Multivariable Cox regression analysis including age, sex, extent of resection, KPS, baseline steroid medication and treatment arm was performed to validate the findings. Significance level was set to alpha ≤ 0.05 and all analyses were two-sided. Statistical analyses were carried out with R (version 4.0.3, The R Foundation for Statistical Computing, https://www.r-project.org, package survminer).

## Results

BMI was unknown in two cases, resulting in 297 patients included in this analysis. 22.5% (67/297) of patients were obese (BMI ≥ 30 kg/m^2^). The median age of the cohort was 56 years (IQR 49–63); 58 years (IQR 50–63) for MGMT methylated and 56 years (IQR 48–63) for MGMT unmethylated patients. Further characteristics and outcome of the included studies have been published previously [10, 12]. Baseline characteristics were similar between obese and non-obese patients (Table 1).

**Table 1:** Baseline characteristics

	All patients			MGMT unmethylated			MGMT methylated		
	Non-obese n = 230	Obese n = 67	p	Non-obese n = 130	Obese n = 38	p	Non-obese n = 100	Obese n = 29	p
BMI, median (IQR)	24.6 (22.7–26.8)	32.3 (31.0–34.2)	< 0.001	24.5 (22.6–27.0)	32.1 (31.0–33.9)	< 0.001	25.0 (22.8, 26.8)	33.3 (31.2–35.2)	< 0.001
Standard treatment arm*(%)	88 (38.3)	29 (43.3)	0.48	44 (33.8)	10 (26.3)	0.43	44 (44.0)	19 (65.5)	0.06
Age, mean (SD)	55.6 (10.3)	56.9 (8.7)	0.37	55.6 (10.8)	55.9 (8.4)	0.86	55.7 (9.5)	58.1 (9.1)	0.22
Male sex (%)	148 (64.3)	42 (62.7)	0.89	87 (66.9)	26 (68.4)	1.0	61 (61.0)	16 (55.2)	0.67
KPS, median (IQR)	90 (90–100)	90 (90–100)	0.15	90 (90–100)	90 (82.5–100)	0.22	95 (90–100)	90 (90–100)	0.42
Baseline steroid (%)	37 (16.1)	14 (20.9)	0.36	24 (18.5)	8 (21.1)	0.82	13 (13.0)	6 (20.7)	0.37
Extent of resection (%)			0.55			0.75			0.73
Biopsy	6 (2.6)	0 (0)		2 (1.6)	0 (0)		4 (4.0)	0 (0)	
PR	99 (43.2)	31 (46.3)		63 (48.8)	21 (55.3)		36 (36.0)	10 (34.5)	
CR	124 (54.1)	36 (53.7)		64 (49.6)	17 (44.7)		60 (60.0)	19 (65.5)	
Study = GLARIUS (%)	130 (56.5)	38 (56.7)	1.00	130 (100)	38 (100)	NA	0 (0)	0 (0)	NA

Values represent number of patients unless indicated otherwise

*BMI* body mass index; *CR* complete resection; *IQR* interquartile range; *n* number of patients; *KPS* Karnofsky performance score; *PR* partial resection; *SD* standard deviation

*Focal radiotherapy, concomitant daily temozolomide, up to six courses of adjuvant temozolomide

### Entire study cohort

For the entire study cohort, both median OS (obese vs. non-obese: 19.2 (95% CI 16.2–21.9) vs. 23.0 (20.1–26.7) months, p = 0.0014) and PFS (obese vs. non-obese: 8.8 (6.0–11.4) vs. 10.0 (9.2–11.7) months, p = 0.008) were shorter in obese patients. The known prognostic and predictive impact of MGMT methylation is emphasized by a greatly differing median OS (GLARIUS: 17.1 (95% CI 15.8–18.1) months, CeTeG/NOA-09: 33.6 (29.3–47.2) months, p < 0.001) and median PFS (8.6 (95% CI 7.9–9.7) vs. 15.7 (11.5–20.4) months, p < 0.001), thus patients with MGMT methylated (CeTeG/NOA-09) and unmethylated tumors (GLARIUS) were subsequently analyzed separately.

### Patients with MGMT unmethylated glioblastoma

**Fig. 1:**
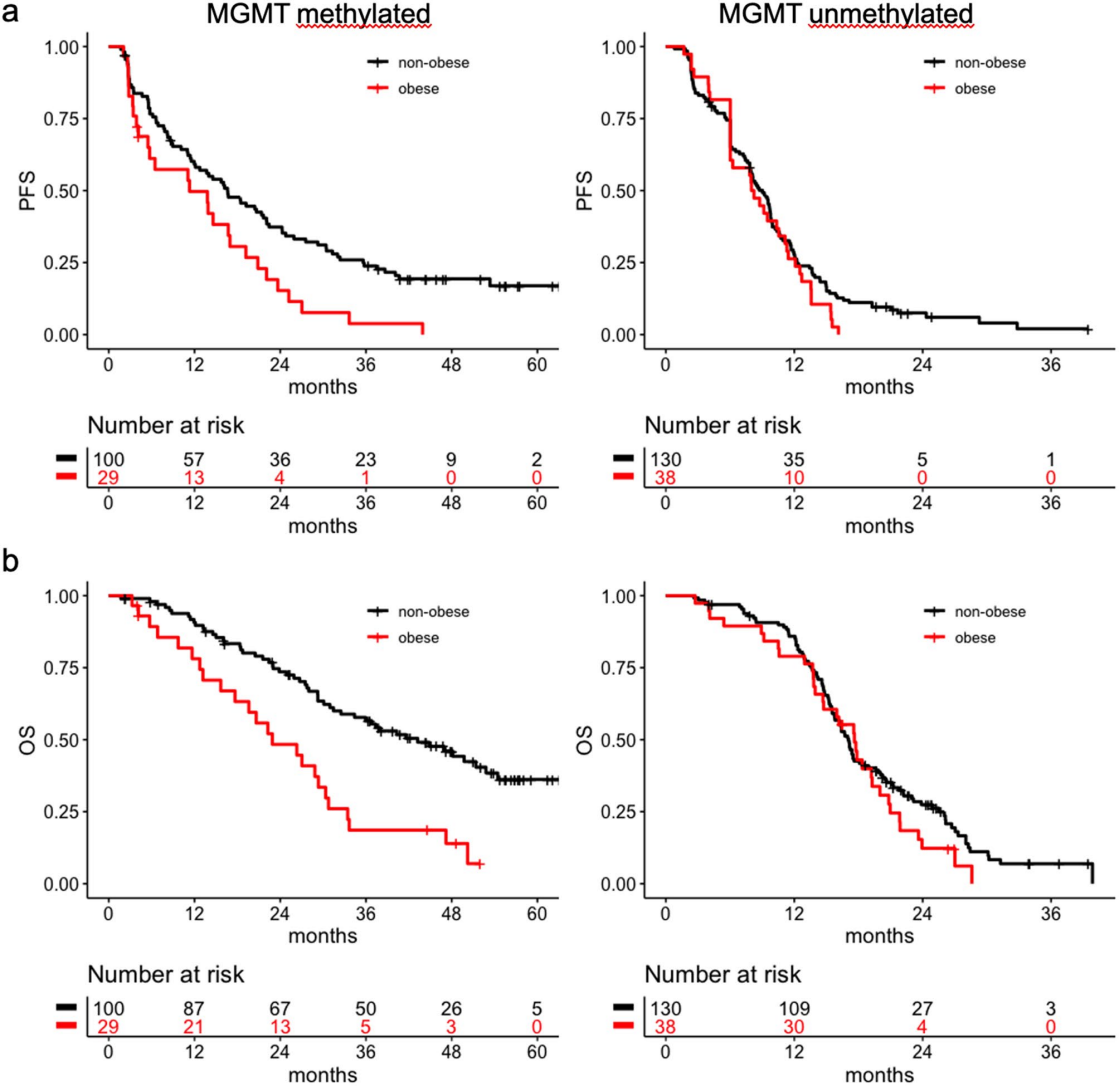
Overall survival and progression-free survival of obese and non-obese patients with MGMT methylated and unmethylated glioblastoma. **a** Progression-free survival of MGMT methylated (left panel) and unmethylated (right panel) glioblastoma patients. **b** Overall survival of MGMT methylated (left) and unmethylated (right) glioblastoma patients. The number of patients at risk is given below each diagram. *MGMT O*6-methylguanine-DNA methyltranferase; *OS* overall survival; *PFS* progression-free survival

In patients with MGMT unmethylated glioblastoma, PFS (obese vs. non-obese: 8.1 (95% CI 6.0–11.3) vs. 9.0 (7.9–9.8) months, p = 0.23; Fig. 1a) and OS (obese vs. non-obese: 17.6 (14.7–20.8) vs. 17.1 (15.8–18.9) months, p = 0.26; Fig. 1b) did not differ between obese and non-obese patients (Fig. 1).

### Patients with MGMT methylated glioblastoma

Among MGMT methylated patients, obesity was associated with shorter PFS (obese vs. non-obese: 11.3 (5.5–20.9) vs. 16.6 (12.1–22.1) months, p = 0.007; Fig. 1a) and OS (obese vs. non-obese: 22.9 (17.7–30.8) vs. 43.2 (32.5–54.4) months, p = 0.001, Fig. 1b). Restricting the analysis to patients with known IDH wildtype status (n = 103) confirmed the findings (p = 0.0208 for PFS and p = 0.0011 for OS). Furthermore, hematotoxicity CTCAE grade 3 or higher occurred with similar frequency in obese and non-obese patients (44.8 vs. 57.0%, p = 0.29).

### Multivariate analysis

Multivariable Cox regression analysis including age, sex, extent of resection, KPS, baseline steroid medication, and treatment arm as covariates confirmed obesity as an independent negative predictor of PFS and OS in MGMT methylated glioblastoma (adjusted odds ratio (aOR) for PFS: 1.95 (95% CI 1.21–3.14), p = 0.007; aOR for OS: 2.57 (1.53–4.31), p < 0.001; Table 2), but not in MGMT unmethylated glioblastoma (aOR for PFS: 1.28 (0.78–1.87), p = 0.20; aOR for OS: 1.17 (0.78–1.75), p = 0.44). Sensitivity analyses restricted to IDH wildtype tumors confirmed these findings for both MGMT methylated glioblastoma (aOR for PFS: 1.78 (1.06–3.00), p = 0.029; aOR for OS: 2.43 (1.39–4.24), p = 0.002) and MGMT unmethylated glioblastoma (aOR for PFS: 0.91 (0.58–1.41), p = 0.66; aOR for OS: 1.46 (0.91–2.35), p = 0.11) [13].

**Table 2:** Multivariate analysis identifies obesity as a negative predictor for overall and progression-free survival in MGMT methylated newly-diagnosed glioblastoma

OS	Adjusted odds ratio	95% CI	p
Obese (vs. non-obese)	2.57	1.53–4.31	< 0.001
Partial resection (vs. biopsy)	0.96	0.25–3.62	0.10
Complete resection (vs. biopsy)	0.76	0.22–2.70	0.67
KPS (per 10% increment)	0.76	0.56–1.01	0.06
Age (per year increment)	1.03	1.00–1.06	0.03
Baseline steroid medication	1.15	0.62–2.14	0.64
Male sex (vs. female)	1.17	0.71–1.92	0.53
TMZ arm (vs. CCNU/TMZ)	0.84	0.51–1.38	0.49

PFS	Adjusted odds ratio	95% CI	p
Obese (vs. non-obese)	1.95	1.21–3.14	0.007
Partial resection (vs. biopsy)	0.54	0.16–1.85	0.33
Complete resection (vs. biopsy)	0.41	0.13–1.33	0.14
KPS (per 10% increment)	9.83	0.65–1.04	0.11
Age (per year increment)	1.02	0.99–1.04	0.10
Baseline steroid medication	0.87	0.47–1.57	0.64
Male sex (vs. female)	0.98	0.64–1.49	0.91
TMZ arm (vs. CCNU/TMZ)	0.83	0.54–1.28	0.40

*CI* confidence interval; *CCNU* lomustine; *IDH* isocitrate dehydrogenase; *KPS* Karnofsky performance score, *OS* overall survival; *PFS* progression-free survival; *TMZ* temozolomide

## Discussion

This analysis of two study cohorts provides evidence for a negative prognostic impact of obesity in MGMT-methylated glioblastoma, but not in MGMT-unmethylated glioblastoma.

We have previously reported that in elderly and frail patients with glioblastoma (median age 72, range 65–86); median KPS 80%, range 50–100), obesity was associated with improved survival [4]. Although these results may seem contradicting, they are in line with the known survival benefit of obesity in elderly [14] and frail patients [15] suffering from different diseases such as diabetes [16], heart failure [17], and metastatic cancer diseases [18] among others. In comparison to this cohort, the study populations of CeTeG/NOA-09 and GLARIUS had favorable baseline characteristics with younger age (median 56 years), a high rate of complete resections (53.8%) and high KPS (median 90%), suggesting sufficient fitness to endure the burden of surgery and radiochemotherapy [10, 12]. These beneficial features might also contribute to the observed median OS of 43 months for the subgroup of non-obese patients with MGMT methylated glioblastoma, comparing favorably to a recent study on the use of immune checkpoint inhibitors in MGMT methylated glioblastoma [19].

The finding of a negative effect of obesity on OS, at least in MGMT-methylated patients, is in line with a previous publication showing an association of obesity with reduced OS in a large retrospective case–control study [9], but inconsistent with results from a recent meta-analysis [20]. However, studies that reported no or even a favorable association of obesity with OS had aspects that make it difficult to compare their results to the findings of the GLARIUS and CeTeG/NOA-09 trial cohort reported here: Jones et al. included patients from 1991 to 2008 who mostly did not receive first-line chemotherapy [7], and three other studies mostly included patients with inferior prognostic factors such as low KPS and/or low complete resection rates [3, 5, 8]. One study that found a positive correlation of obesity and OS is difficult to interpret since obese and non-obese patients were imbalanced regarding percentage of complete resections (68.8% vs. 55.2%) and female patients (66% vs. 35%) [6]. Female sex may be a favorable prognostic factor that was not included in univariate and multivariate analyses [21]. Considering all available data, the best hypothesis regarding the association of obesity and OS would be that in patients with inferior prognostic factors such as comparably low performance status and even more in elderly and frail patients, obesity may have a positive impact, while in patients with favorable prognostic factors (e.g. populations in clinical trials) obesity may be a negative prognostic factor, especially in the context of effective, survival-prolonging chemotherapy. The survival benefit of obesity in oncology, termed obesity paradox, might be explained by the inadequacy of BMI to measure body fat in cancer patients undergoing weight changes, as it does not distinguish adipose and muscle tissue [22]. Indeed, skeletal muscle status is an independent prognostic parameter in glioblastoma [20, 23], and obese patients have on average higher levels of muscle. Therefore, the obesity paradox might be most significant in elderly, frail or dependent patients, where sarcopenia is frequent. On the other hand, it is absent in our trial cohort (with comparably favorable prognostic factors), resulting from the assumed relative absence of sarcopenia, and potentially detrimental effects of adipose tissue on glioblastoma treatment might be demasked. Future studies considering body composition might contribute to solving this interesting dichotomy.

The mechanistic link between survival and obesity remains elusive, as no death was related to obesity itself in the CeTeG/NOA-09 trial (unknown: 2 cases). Obesity is linked to reduced glucose sensitivity and increased blood glucose levels, a known risk factor in glioblastoma [24, 25]. HbA1c and glucose levels were not available in our cohorts, but previous data suggests an independent prognostic effect of diabetes mellitus and obesity [8, 9]. Recently, an obesity-inducing high-fat diet was described to promote aggressive disease with shortened survival via intracerebral fat accumulation and impaired hydrogen sulfide production leading to increased proliferation and chemotherapy resistance in glioblastoma [26]. Furthermore, obesity is inversely correlated with socioeconomic status, a known prognostic factor for survival in glioblastoma [27, 28].

Of note, obesity was associated with shorter survival in MGMT methylated, but not in MGMT unmethylated tumors. While it is possible that the shorter overall survival in MGMT unmethylated tumors impeded detection of a survival difference between obese and non-obese patients, an alternative mechanistic hypothesis seems promising: MGMT promoter methylation reduces MGMT expression, an enzyme removing alkyl groups from the *O*^6^ position of guanine [29]. These lesions trigger cytotoxicity and apoptosis in a process requiring a functioning mismatch repair pathway and DNA damage signaling by ATR and ATM [29]. Elevated fatty acid levels were reported to compromise the induction of p21 downstream of ATM [30], which is required for temozolomide sensitivity [31]. Similarly, increased levels of free fatty acids lead to mitochondrial DNA damage culminating in cellular apoptosis induction [32, 33]. A recent study revealed that the combinatory treatment with the glycolytic inhibitor dichloracetate and the partial fatty acid oxidation inhibitor ranolazine yielded reduced colony forming activity and apoptosis of glioblastoma cells in vitro [31]. Murine in vivo experiments under this combination treatment resulted in increased median survival [34], supporting the proposed mechanistic link to reflect an intratumoral cellular effect. Thus, elevated fatty acid levels in obese patients might compromise the therapeutic response to alkylating chemotherapy in MGMT methylated glioblastoma. In MGMT unmethylated glioblastoma, on the other hand, the benefit of temozolomide is at best limited, rendering this effect negligible [35].

## Conclusions

We conclude that obesity might be a prognostic marker in newly diagnosed MGMT-methylated but not MGMT-unmethylated glioblastoma. If confirmed by further analyses, it might inform patient stratification in future trials and enable individual prognostication and informed decision-making.

## Data Availability

Restrictions apply to the availability of these data due to privacy restrictions.

## References

[1] Wen PY, Weller M, Lee EQ (2020). Glioblastoma in adults: a Society for Neuro-Oncology (SNO) and European Society of Neuro-Oncology (EANO) consensus review on current management and future directions. Neuro-Oncology.

[2] Weller M, van den Bent M, Preusser M (2021). EANO guidelines on the diagnosis and treatment of diffuse gliomas of adulthood. Nat Rev Clin Oncol.

[3] Potharaju M, Mangaleswaran B, Mathavan A (2018). Body mass index as a prognostic marker in glioblastoma multiforme: a clinical outcome. Int J Radiat Oncol Biol Phys.

[4] Schneider M, Potthoff A-L, Scharnböck E (2020). Newly diagnosed glioblastoma in geriatric (65+) patients: impact of patients frailty, comorbidity burden and obesity on overall survival. J Neurooncol.

[5] Cha J-Y, Park J-S, Hong Y-K (2021). Impact of body mass index on survival outcome in patients with newly diagnosed glioblastoma: a retrospective single-center study. Integr Cancer Ther.

[6] Valente Aguiar P, Carvalho B, Vaz R, Linhares P (2021). Body mass index as an independent prognostic factor in glioblastoma. Cancer Causes Control.

[7] Jones LW, Ali-Osman F, Lipp E (2010). Association between body mass index and mortality in patients with glioblastoma mutliforme. Cancer Causes Control.

[8] Chambless LB, Parker SL, Hassam-Malani L (2012). Type 2 diabetes mellitus and obesity are independent risk factors for poor outcome in patients with high-grade glioma. J Neurooncol.

[9] Siegel EM, Nabors LB, Thompson RC (2013). Prediagnostic body weight and survival in high grade glioma. J Neurooncol.

[10] Herrlinger U, Tzaridis T, Mack F (2019). Lomustine-temozolomide combination therapy versus standard temozolomide therapy in patients with newly diagnosed glioblastoma with methylated MGMT promoter (CeTeG/NOA-09): a randomised, open-label, phase 3 trial. Lancet.

[11] Tzaridis T, Schäfer N, Weller J (2021). MGMT promoter methylation analysis for allocating combined CCNU/TMZ chemotherapy: lessons learned from the CeTeG/NOA-09 trial. Int J Cancer.

[12] Herrlinger U, Schäfer N, Steinbach JP (2016). Bevacizumab plus irinotecan versus temozolomide in newly diagnosed O^6^-methylguanine-DNA methyltransferase nonmethylated glioblastoma: the randomized GLARIUS trial. J Clin Oncol.

[13] Johnson RM, Phillips HS, Bais C (2020). Development of a gene expression-based prognostic signature for IDH wild-type glioblastoma. Neuro-Oncology.

[14] Lee JSW, Auyeung T-W, Chau PPH (2014). Obesity can benefit survival-a 9-year prospective study in 1614 Chinese nursing home residents. J Am Med Dir Assoc.

[15] Lee Y, Kim J, Han ES (2014). Frailty and body mass index as predictors of 3-year mortality in older adults living in the community. Gerontology.

[16] Bijani A, Cumming RG, Hosseini S-R (2018). Obesity paradox on the survival of elderly patients with diabetes: an AHAP-based study. J Diabetes Metab Disord.

[17] Padwal R, McAlister FA, McMurray JJV (2014). The obesity paradox in heart failure patients with preserved versus reduced ejection fraction: a meta-analysis of individual patient data. Int J Obes (Lond).

[18] Pamoukdjian F, Aparicio T, Canoui-Poitrine F (2019). Obesity survival paradox in cancer patients: results from the physical frailty in older adult cancer patients (PF-EC) study. Clin Nutr.

[19] Lim M, Weller M, Idbaih A et al (2022) Phase 3 trial of chemoradiotherapy with temozolomide plus nivolumab or placebo for newly diagnosed glioblastoma with methylated MGMT promoter. Neuro-Oncology. 10.1093/neuonc/noac116 10.1093/neuonc/noac116 PMC9629431 35511454

[20] Guven DC, Aksun MS, Cakir IY (2021). The association of BMI and sarcopenia with survival in patients with glioblastoma multiforme. Future Oncol.

[21] Ostrom QT, Rubin JB, Lathia JD (2018). Females have the survival advantage in glioblastoma. Neuro-Oncology.

[22] Caan BJ, Cespedes Feliciano EM, Kroenke CH (2018). The importance of body composition in explaining the overweight paradox in cancer-counterpoint. Cancer Res.

[23] Furtner J, Genbrugge E, Gorlia T (2019). Temporal muscle thickness is an independent prognostic marker in patients with progressive glioblastoma: translational imaging analysis of the EORTC 26101 trial. Neuro-Oncology.

[24] Derr RL, Ye X, Islas MU (2009). Association between hyperglycemia and survival in patients with newly diagnosed glioblastoma. J Clin Oncol.

[25] Lu VM, Goyal A, Vaughan LS, McDonald KL (2018). The impact of hyperglycemia on survival in glioblastoma: a systematic review and meta-analysis. Clin Neurol Neurosurg.

[26] Silver DJ, Roversi GA, Bithi N (2021). Severe consequences of a high-lipid diet include hydrogen sulfide dysfunction and enhanced aggression in glioblastoma. J Clin Investig.

[27] Tosoni A, Gatto L, Franceschi E (2021). Association between socioeconomic status and survival in glioblastoma: an Italian single-centre prospective observational study. Eur J Cancer.

[28] Schmidt LS, Nielsen H, Schmiedel S, Johansen C (2008). Social inequality and incidence of and survival from tumours of the central nervous system in a population-based study in Denmark, 1994–2003. Eur J Cancer.

[29] Kaina B, Christmann M (2019). DNA repair in personalized brain cancer therapy with temozolomide and nitrosoureas. DNA Repair (Amst).

[30] Zeng L, Wu G-Z, Goh KJ (2008). Saturated fatty acids modulate cell response to DNA damage: implication for their role in tumorigenesis. PLoS ONE.

[31] Aasland D, Götzinger L, Hauck L (2019). Temozolomide induces senescence and repression of DNA repair pathways in glioblastoma cells via activation of ATR-CHK1, p21, and NF-κB. Cancer Res.

[32] Grishko V, Rachek L, Musiyenko S (2005). Involvement of mtDNA damage in free fatty acid-induced apoptosis. Free Radic Biol Med.

[33] Rachek LI, Musiyenko SI, LeDoux SP, Wilson GL (2007). Palmitate induced mitochondrial deoxyribonucleic acid damage and apoptosis in l6 rat skeletal muscle cells. Endocrinology.

[34] McKelvey KJ, Wilson EB, Short S (2021). Glycolysis and fatty acid oxidation inhibition improves survival in glioblastoma. Front Oncol.

[35] Hegi ME, Diserens A-C, Gorlia T (2005). MGMT gene silencing and benefit from temozolomide in glioblastoma. N Engl J Med.

